# A new bioinformatics approach identifies overexpression of GRB2 as a poor prognostic biomarker for prostate cancer

**DOI:** 10.1038/s41598-021-85086-9

**Published:** 2021-03-11

**Authors:** Teppei Iwata, Anna S. Sedukhina, Manabu Kubota, Shigeko Oonuma, Ichiro Maeda, Miki Yoshiike, Wataru Usuba, Kimino Minagawa, Eleina Hames, Rei Meguro, Sunny Cho, Stephen H. H. Chien, Shiro Urabe, Sookhee Pae, Kishore Palanisamy, Toshio Kumai, Kazuo Yudo, Eiji Kikuchi, Ko Sato

**Affiliations:** 1 Department of Frontier Medicine, Institute of Medical Science, Graduate School of Medicine, St. Marianna University, Kawasaki, 2168511 Japan; 2 Department of Urology, School of Medicine, St. Marianna University, Kawasaki, 2168511 Japan; 3 Department of Pathology, School of Medicine, St. Marianna University, Kawasaki, 2168511 Japan; 4 Department of Pathology, Kitasato University Kitasato Institute Hospital, Minato, 1080072 Japan; 5 Department of Pathology, Kitasato University School of Medicine, Sagamihara, 2520374 Japan; 6 Department of Pharmacogenomics, Graduate School of Medicine, St. Marianna University, Kawasaki, 2168511 Japan; 7 K International School Tokyo, Koto, 1350021 Japan

**Keywords:** Medical research, Gene regulatory networks

## Abstract

A subset of prostate cancer displays a poor clinical outcome. Therefore, identifying this poor prognostic subset within clinically aggressive groups (defined as a Gleason score (GS) ≧8) and developing effective treatments are essential if we are to improve prostate cancer survival. Here, we performed a bioinformatics analysis of a TCGA dataset (GS ≧8) to identify pathways upregulated in a prostate cancer cohort with short survival. When conducting bioinformatics analyses, the definition of factors such as “overexpression” and “shorter survival” is vital, as poor definition may lead to mis-estimations. To eliminate this possibility, we defined an expression cutoff value using an algorithm calculated by a Cox regression model, and the hazard ratio for each gene was set so as to identify genes whose expression levels were associated with shorter survival. Next, genes associated with shorter survival were entered into pathway analysis to identify pathways that were altered in a shorter survival cohort. We identified pathways involving upregulation of GRB2. Overexpression of GRB2 was linked to shorter survival in the TCGA dataset, a finding validated by histological examination of biopsy samples taken from the patients for diagnostic purposes. Thus, GRB2 is a novel biomarker that predicts shorter survival of patients with aggressive prostate cancer (GS ≧8).

## Introduction

Prostate cancer, the most common cancer in males, caused the second highest number of cancer-related deaths in the USA in 2018^1^. A subset of prostate cancer displays an aggressive clinical course^2^. The clinical outcome of prostate cancer is strongly associated with the Gleason Score (GS), which is a grading system based on morphological characteristics. Histological grades are scored from 1 (resembles normal tissue) to 5 (far from normal tissue). The Gleason score is calculated as the sum of the primary histological grade (the most predominant grade) and the secondary histological grade (the second most predominant grade)^3^. A high GS is defined as high-risk^4,5^. However, extensive studies show that a lower GS in those with high-risk prostate cancer is often linked to relatively favorable outcome^6^. These data indicate that further subdivision is required to provide a better estimate of prognosis. Therefore, identifying biomarkers specific for the aggressive subset of prostate cancer is essential. Also, we need to develop effective treatments for these poor prognostic subsets if we are to improve overall clinical outcomes.

Bioinformatics analysis is a versatile tool for identifying biomarkers linked to shorter survival; these biomarkers can then be a targeted to develop novel treatments. However, there are several pitfalls associated with bioinformatics analysis. For example, accurate definition of terms (e.g., “shorter” or “longer survival” and “over- “ or under-expression”) is required. However, there is no way of knowing whether these definitions are correct, and inappropriate definitions can affect the results. Therefore, to obtain reliable candidates from bioinformatics analysis, we removed all subjective definitions and used cutoff points determined by statistical analysis. Then, we investigated whether expression of the biomarker(s) identified by bioinformatics analysis was associated with histological diagnoses in biopsy section from a shorter survival clinical cohort.

GRB2 plays a role in signal transduction by oncogenic tyrosine kinases, resulting in activation of Ras-mitogen-activated protein kinases^7^. Tyrosine kinase signaling plays a pivotal role in cancer development and progression; therefore, expression of components in these signaling pathways has been well-studied. For example, increased expression of GRB2 is linked to poor survival in patients with gastric cancer^8^, and overexpression of GRB2 is associated with lymph node metastasis and shorter survival in patients with esophageal cancer^9^. Furthermore, overexpression of PASMD14, a deubiquitinase that protects GRB2 against proteasomal degradation, resulting in increased expression of GRB2, is linked to tumor growth and metastasis in patients with hepatocellular carcinoma^10^. Here, we show that overexpression of GRB2 is linked to shorter survival of patients with aggressive prostate cancer (defined as GS ≧8).

## Results

### Identification of pathways upregulated in the short survival cohort

Prostate cancer with a GS ≥ 8 is defined as high-risk^11^; therefore, to identify pathways and molecules associated with shorter survival in a cohort with a poor clinical outcome, we selected patients with a GS ≥ 8 from the TCGA dataset (Table 1). Patients without survival data were excluded. The TCGA sub-cohort included 201 cases, all with a localized tumor (i.e., no metastatic spread). All the subjects had undergone radical prostectomy (RP), and 50 had undergone radiotherapy (RT). Of the 201 cases, 17 lacked RT data. Most cases who underwent RT had a residual tumor. Of the 201 cases, three lacked data about residual tumor. When calculating survival hazard ratios (HRs) for patients overexpressing certain molecules, it is important to accurately define overexpression to remove the possibility of bias. To define the cutoff point used in this study, we used an algorithm that identified the best cutoff value based on data from a Cox proportional hazard model^12^. The method is based on division of a given set of events into two subsets or groups by selecting an appropriate “cutoff”, C = {C1, C2, …, Ck}, which is a non-linear function defined to maximize statistical differences (assessed using the log-rank test) between two groups. The recurrence-free survival HRs of all genes (n = 18,393) expressed at levels above the cutoff value defined by the algorithm were calculated, and 4956 genes were identified as being associated with survival (*p* value < 0.05). The number of genes was reduced by calculating the z-score. If the z-score of the cutoff value was close to zero, cutoff value is near the average value in the cohort. To identify genes whose “over” or “under” expression leads to shorter survival, those genes with a cutoff value z-score ≥ 1 (overexpression) or ≤ -1 (under-expression) were picked up. Thus, we identified 769 genes as being associated with survival. Of these, 613 and 156 were linked to shorter survival and longer survival, respectively. The raw data are presented in Supplementary Table S1. The 613 linked to shorter survival were entered into pathway analysis using IPA software to identify pathways that were up- or down-regulated in the shorter survival cohort. Pathway analysis revealed that 11 pathways were upregulated significantly (defined by an activation z-score ≥ 1 and a *p* value < 0.05) in the shorter survival cohort (Table 2). GRB2 plays a role in the majority of these pathways (10 out of 11), suggesting that GRB2 is a central player in pathways upregulated in the shorter survival cohort (Table 2). Therefore, we focused on the GRB2 gene.

**Table 1:** Baseline characteristics of patients in the TCGA database (Gleason score ≥ 8).

Age		T category		Gleason score		Histology	
Average (y)	62.4	pT2b	3	3 + 5	7	Adenocarcinoma	145
Range	44–78	pT2c	24	4 + 4	50	Aca mixed subtype	2
		pT3a	62	5 + 3	7	Mucinous ca	1
		pT3b	103	4 + 5	97	Signet ring cell ca	1
		pT4	7	5 + 4	37	Infiltrating duct ca	8
		NA	2	5 + 5	3	Acinar cell ca	44

The numbers to the right of each column denote the number of patients. pT: pathological T; NA: not applicable; Adenocarcinoma: Adenocarcinoma NOS; Aca: Adenocarcinoma; Ca: carcinoma.

**Table 2:** Pathways upregulated in the shorter survival cohort.

Pathway	*p* value	z-score	Molecule
Oncostatin M signaling	0.0034	1.342	IL6ST, GRB2, OSM, HRAS, PLAU
eNOS signaling	0.0048	1.667	CALM1, PRKAR2B, CHRNA6, GRB2, PIK3R1, VEGFB, AQP1, CHRM1, CNGA3, PRKD1, CHRM5
ErbB4 signaling	0.0148	1.000	GRB2, PIK3R1, APH1A, HRAS, PSEN2, PRKD1
BMP signaling pathway	0.0185	1.633	PRKAR2B, BMPR1A, GRB2, MAPK8, HRAS, BMP6
Mouse ES cell pluripotency	0.0249	1.134	TP53, IL6ST, GRB2, BMPR1A, PIK3R1, HRAS, DVL3
NOS in the CV system	0.0260	1.134	CALM1, PRKAR2B, GRB2, PIK3R1, VEGFB, CHRM1, PRKD1
EGF signaling	0.0299	1.342	CSNK2A1, GRB2, PIK3R1, MAPK8, HRAS
Gβγ signaling	0.0345	1.890	PRKAR2B, CAV2, GNAT1, GRB2, HRAS, CACNG8, PRKD1
UVC-Induced MAPK signaling	0.0362	1.000	TP53, MAPK8, HRAS, PRKD1
PDGF signaling	0.0365	1.633	CSNK2A1, GRB2, PIK3R1, MAPK8, HRAS, INPPL1
Ceramide signaling	0.0415	1.342	PPP2R1A, GRB2, PIK3R1, MAPK8, HRAS, NSMAF

*ES cell* embryonic stem cell, *NOS* nitric oxide synthase, *CV* cardiovascular.

### Effect of GRB2 expression on survival of prostate cancer patients

**Figure 1:**
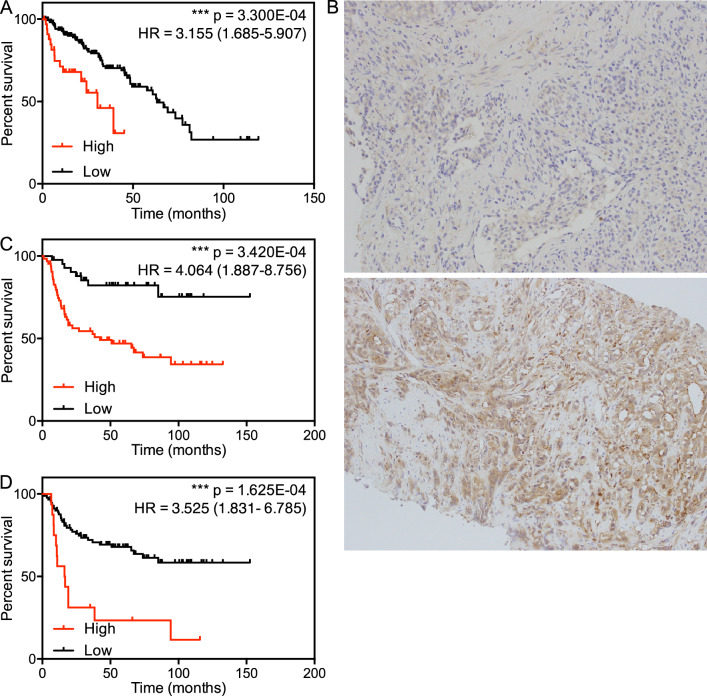
Effect of GRB2 expression on survival. (**A**) Recurrence-free survival curves for prostate cancer patients in the TCGA dataset. Each population was divided into two groups according to the cutoff point defined by Cox regression model (cutoff z-score = 1.0951). (**B**) Representative images of prostate cancer biopsy samples showing low (top) or high (bottom) expression of GRB2 protein. C and D. Recurrence-free survival curves for prostate cancer patients in the local clinical cohort. Each population was divided into two groups according to the cutoff point defined by the Cox regression model (cutoff H-score = 69.8592) (**C**) and a z-score = 1 (**D**).

Next, we examined the TCGA dataset to ascertain whether expression of GRB2 mRNA affects prostate cancer survival. The TCGA cohort was divided according to the threshold defined by the algorithm and then analyzed using the Kaplan–Meier method. The cohort overexpressing GRB2 showed shorter recurrence-free survival; indeed, the average time to recurrence in the groups with or without GRB2 overexpression was 12.08 and 31.91 months, respectively (HR = 3.155; 95% confidence interval (CI): 1.685–5.907) (Fig. 1, panel A). To eliminate the effect of factors such as age, GS, and T category, we performed multivariate analysis, which identified overexpression of GRB2 is an independent prognostic factor for shorter survival (HR = 2.410; 95% CI: 1.080–5.379) (Table 3). To examine the effect of GRB2 protein expression on survival, we investigated biopsy samples taken at the time of initial diagnosis at St. Marianna University Hospital between 2004 and 2014 (Table 4). Our clinical cohort included 107 cases of both localized and metastatic disease. Localized cases underwent either RP or RT, and all metastatic cases received hormonal therapy. Of the 22 cases who underwent RP, 11 had residual tumor; none of the 11 cases received additional RT. Prior to analyses, we examined the correlation between GRB2 mRNA and protein expression using a dataset in which paired expression of mRNA and protein was examined in 29 tissues^13^. This analysis revealed a positive correlation between GRB2 mRNA and protein expression (Pearson r = 0.7676, *p* < 0.0001; Supplementary Fig. S3).

**Table 3:** Multivariate analysis of the effect of GRB2 expression on survival (based on data from the TCGA).

	Unadjusted		Adjusted		
	HR (95%CI)	*p* value	HR (95%CI)	*p* value	*p* value^a^
GRB2	3.225 (1.723–6.034)	2.500e−04***	3.144 (1.643–6.018)	2.063e−03***	1.000e−03***
Age	1.007 (0.970–1.045)	7.182e−01	1.016 (0.977–1.056)	4.278e−01	1.000e+00
GS	1.179 (1.003–1.387)	4.556e−02*	1.136 (0.962–1.340)	1.325e−01	1.822e−01
T category	1.367 (1.007–1.856)	4.490e−02*	1.226 (0.895–1.680)	2.039e−01	1.796e−01

GS: Gleason score: “*” and “***” indicate *p* value < 0.05 and < 0.001, respectively.^a^ Adjusted *p* value (using a Bonferroni correction).

**Table 4:** Baseline characteristics of the samples from the clinical cohort.

Age		72 (55–86)*
cT category	T1c	26
	T2a	8
	T2b	11
	T2c	21
	T3a	18
	T3b	13
	T4	9
	NA	1
Gleason score	3 + 5	3
	5 + 3	3
	4 + 4	55
	4 + 5	27
	5 + 4	13
	5 + 5	6
Histology	Adenocarcinoma NOS	107
Treatment	RP	22
	RT	45
	HT	40

*Age is shown as the median (range). All other data represent the number of patients. cT: clinical T; NA: not available; NOS: not otherwise specified; RP: radical prostatectomy; RT: radiation therapy; HT: hormonal therapy.

Next, we tested the specificity of the anti-GRB2 antibody. Staining of U2OS cell lines showing over- or under-expression of GRB2 revealed that the antibody detects GRB2 specifically (Supplementary Fig. S1). We found that high expression of GRB2 protein was observed only in cancer cells, although it varied widely between samples (Fig. 1, panel B). Protein expression was measured using the IHC profiler plugin in ImageJ with high magnification/resolution images to eliminate the effect of GRB2 expression in stromal cells^14^. We further undertook a study of GRB2 expression in stromal cells using an image containing both cancer and stromal cells. High expression of GRB2 was detected only in cancer cells, whereas only weak expression was observed in stromal cells (Supplementary Fig. S2), suggesting that GRB2 is expressed mainly by cancer cells. We investigated the effect of GRB2 expression on time to recurrence that was defined according to the European Association of Urology criteria (https://uroweb.org/guideline/prostate-cancer/): PSA > 0.2 ng/mL and rising after RP; PSA > nadir + 2 ng/mL after RT; and PSA rising, with serum testosterone < 50 ng/mL, after hormonal therapy. According to the best cutoff value calculated by the Cox regression model, increased expression of GRB2 was linked to shorter recurrence-free survival: the average time to recurrence in groups with or without GRB2 overexpression was 2.33 and 22.24 months, respectively (HR = 4.064; 95% CI: 1.887–8.756) (Fig. 1, panel C). When we conducted multivariate analysis adjusting for age, iPSA, GS, and T category, GRB2 was identified as a prognostic factor for a poor clinical outcome (HR = 3.434; 95% CI: 1.567–7.528) (Table 5). The best cutoff value calculated by the Cox regression model based on the TCGA dataset was an mRNA expression z-score = 1.0951; however the best cutoff value based on data from the clinical cohort was a protein expression z-score of -0.3846, suggesting that the threshold for the definition of overexpression is below the mean value. Overexpression is often defined as a z-score ≥ 1. Therefore, we examined the effect of GRB2 protein overexpression (defined as z-score ≥ 1) on survival. We identified three classes of GRB2 expression defined by z-scores, > 1.095 in TCGA (18.91%), > -0.3846 in clinical practice (59.43%) and > 1 in clinical practice (15.09%). This is suggestive of a strong predictive value for GRB2 expression linked to its z-score that is invariant of using either GRB2 mRNA or protein levels for z-score calculation. Overexpression of GRB2 protein, defined as a z-score ≥ 1, was also linked to shorter recurrence-free survival: the average time to recurrence in groups with or without GRB2 overexpression was 20.47 and 24.77 months, respectively (HR = 3.525; 95% CI: 1.831–6.785) (Fig. 1, panel D). The effect of GRB2 protein overexpression remained significant in multivariate analysis (HR = 2.058; 95% CI: 1.004–4.215) (Table 6). Taken together, these data suggest that overexpression of GRB2 at both the mRNA and protein level is linked to shorter survival of those with aggressive prostate cancer.

**Table 5:** Multivariate analysis of the effect of GRB2 expression on survival (based on data from clinical samples).

	Unadjusted		Adjusted		
	HR (95%CI)	*p* value	HR (95%CI)	*p* value	*p* value^a^
GRB2	3.985 (1.846–8.601)	4.282e−04***	3.434 (1.567–7.528)	2.063e−03***	2.141e−03***
Age	0.999 (0.952–1.048)	9.564e−01	0.991 (0.943–1.041)	7.078e−01	1.000e+00
iPSA	1.000 (1.000–1.000)	2.094e−02*	1.000 (0.999–1.041)	7.500e−01	1.047e−01
GS	1.085 (0.930–1.266)	2.999e−01	0.128 (0.965–1.320)	1.311e−01	1.000e−00
T category	1.559 (1.314–1.849)	3.449e−07***	1.561 (1.312–1.856)	4.775e−07***	1.725e−06***

GRB2 cut off, H-score ≥ 69.8532; iPSA: initial PSA; GS: Gleason score; “*” and “***” indicate *p* value < 0.05 and < 0.001, respectively. ^a^Adjusted *p* value (using a Bonferroni correction).

**Table 6:** Multivariate analysis of effect of GRB2 expression on survival (based on data from clinical samples).

	Unadjusted		Adjusted		
	HR (95%CI)	*p* value	HR (95%CI)	*p* value	*p* value^a^
GRB2	3.603 (1.865–6.960)	1.360e−04***	2.058 (1.004–4.215)	4.859e−02*	6.800e−04***
Age	0.999 (0.952–1.048)	9.564e−01	0.992 (0.943–1.045)	7.726e−01	1.000e+00
iPSA	1.000 (1.000–1.000)	2.094e−02*	1.000 (0.999–1.000)	1.859e−01	1.047e−01
GS	1.085 (0.930–1.267)	2.999e−01	0.176 (1.004–1.378)	4.468e−02*	1.000e+00
T category	1.559 (1.314–1.849)	3.449e−07***	1.489 (1.244–1.782)	1.415e−05***	1.725e−06***

GRB2 cut off, z-score ≥ 1; iPSA: initial PSA: GS: Gleason score; “*” and “***” indicate *p* value < 0.05 and < 0.001, respectively. ^a^Adjusted *p* value (using a Bonferroni correction).

## Discussion

In our previous study based on bioinformatics, we identified differentially expressed genes in prostate cancer cohorts showing shorter and longer survival^15^. Shorter or longer survival was defined based on time to recurrence of 1, 3, or 5 years. In this study, molecules showing high expression in the shorter survival cohort (defined as recurrence within 1, 3, and 5 years) were totally different, thereby casting doubt on whether these definitions are appropriate. Therefore, we eliminated this strategy from this study. In our previous study, we also calculated the HR of all the overexpressed genes. The cutoff value used to define overexpression was a z-score of 1. It is clear that the z-score depends on the distribution of expression within the cohort. In other words, the definition of overexpression based on the z-score will be totally different for a different cohort. Indeed, in the present study, the best cutoff value calculated using a Cox regression model was a GRB2 expression z-score of ≥ 1 for the TCGA cohort, but a z-score < 1 for the clinical sample cohort. When we analyzed the effect of overexpression defined as a z-score ≥ 1 in the clinical cohort, we found that overexpression was associated with shorter survival. Therefore, we believe that our conclusion, i.e., that overexpression of GRB2 affects clinical outcome of those with progressive prostate cancer, is reliable. However, repeated analysis using different cohorts is required to confirm the reproducibility of these results.

Receptor tyrosine kinases (RTKs) promote transformation and metastasis by activating Ras/mitogen-activated protein kinase (MAPK) and PI3K/AKT^16^. GRB2 acts as an adaptor protein to activate the signaling cascades. Indeed, overexpression of GRB2 increases RAS and MAPK levels^17^, suggesting that GRB2 plays a main role in the transformation process. In breast cancer, overexpression of Her2 is often observed, which activates the Ras/MAPK and PI3K/AKT pathways and promotes cell proliferation and migration^16^. In cells expressing activated HER2, the dominant negative mutant of GRB2 that inhibits a trimer formation of Shc-GRB2-Sos, suppresses the development of transforming phenotypes through RAS inactivation^18^. In Her2-expressing prostate cancer cells, studies in a xenograft model show that inhibition of GRB2, together with the use of anti-tumor agents, suppresses tumor proliferation^19^. The data presented herein indicate that GRB2 overexpression impacts survival, regardless of HER2 expression. This suggests that GRB2 plays an important role, along with Her2 and other RTK signaling pathways, in the progression of prostate cancer.

We have identified a molecule that plays a major role in pathways upregulated in the short survival cohort; this suggests that the biomarker that we have identified can be used as a target to develop effective treatments. Since GRB2 plays a role in progression of several malignancies, studies have tried to develop a therapy targeting GRB2^8–10^. Inhibiting GRB2 using a liposome-incorporated oligonucleotide to suppress GRB2 protein expression (L-GRB2) inhibits proliferation of BCR-ABL-positive leukemia cells both in vitro and *in vivo*^20^. Furthermore, because duplication of the GRB2 gene is common in leukemias, GRB2 is a possible target for therapeutics. Indeed, administration of L-GRB2 to acute myeloid leukemia patients in a Phase 1/1b trial showed promising results^21^. Therefore, we hypothesized that inhibiting GRB2 is a possible therapeutic option for patients with prostate cancer overexpressing GRB2.

## Methods

### Bioinformatic analysis

Z-scores (RNA seq V2 RSEM), available in cBioPortal (http://www.cbioportal.org), were used to calculate the HR^22^. The cutoff value used to define increased or decreased expression was based on test scores calculated by a Cox regression model^23^. This calculation was made using the “cutp” function of the “survMisc” R package (http://CRAN.R-project.org/package=survival)12.

### Ingenuity pathway analysis (IPA)

IPA was performed as described previously^24,25^. Briefly, molecules associated with shorter survival were analyzed by IPA software to search for canonical pathways. The significance of the canonical pathways was denoted by the calculated *p* value. Statistical significance was considered as a z-score ≥ 1 and a *p* value < 0.05.

### Statistical analysis

HRs and their significance were calculated using a Cox proportional hazard model. The Kaplan–Meier method was used for survival analysis. Multivariate analysis was conducted using Cox proportional hazard models based on the coxph function in the survival library in R. The Adjusted *p* value by Bonferroni correction was calculated using the “p.adjust” function of the “stats version 4.1.0” R package. All other analyses were performed using GraphPad prism software. Differences were considered statistically significant when the two-tailed *p* value was < 0.05.

### Patients

Patients diagnosed with prostate cancer by biopsy at St. Marianna University Hospital from January 2003 to August 2014 were considered for inclusion in the study. Patients without precise information regarding their case, and patients who did not undergo standard therapy due to advanced age or severe comorbidities, were excluded. Of the remaining patients, 107 had a Gleason score ≥ 8 and were assigned to the study cohort. Two pathologists and two urologists examined biopsies and chose appropriate samples based on the size of the region occupied by the cancer cells.

### Study approval

All experiments involving clinical samples were approved by the St. Marianna University clinical ethics committee, and all participants provided informed consent (approval number: 3181). All the experiments were conducted in accordance with ethical guidelines, including the tenets of the Declaration of Helsinki.

### Immunohistochemistry (IHC) and measurement of protein expression

IHC was performed as described previously^26^. Briefly, antigen retrieval in fixed biopsy sections was performed for 40 min in Antigen Retrieval Solution (pH 6.0) (DAKO) heated to 96 °C in a steamer, followed by incubation with an anti-GRB2 antibody for 60 min. Next, samples were incubated with a secondary antibody (Nichirei Biosciences) for 30 min. The antibodies used (and their concentrations) are listed below. To measure protein expression, > 200 cells in each of three high-power fields were evaluated and the H-score was calculated using the IHC profiler plugin in ImageJ^14^. The value for the field with the highest H-score was validated to examine the link with survival.

### Antibodies

An anti-GRB2 antibody (Abcam: ab111031, 1:400) was used for IHC.

## Supplementary Information

Supplementary Information 1.

Supplementary Information 2.
