# Supplementary material for: A new bioinformatics approach identifies overexpression of GRB2 as a poor prognostic biomarker for prostate cancer
Source: Sci Rep. 2021 Mar 11;11:5696. doi: 10.1038/s41598-021-85086-9 (PMC7952695; doi:10.1038/s41598-021-85086-9)
Supplement: Supplementary file 1 — Supplementary Information 1. [file 41598_2021_85086_MOESM1_ESM.pdf]

A new bioinformatics approach identifies overexpression of GRB2 as a poor prognostic biomarker for prostate cancer

Teppei Iwata, Anna S Sedukhina, Manabu Kubota, Shigeko Oonuma, Ichiro Maeda, Miki Yoshiike, Wataru Usuba, Kimino Minagawa, Eleina Hames, Rei Meguro, Sunny Cho, Stephen HH Chien, Shiro Urabe, Sookhee Pae, Kishore Palanisamy, Toshio Kumai, Kazuo Yudo, Eiji Kikuchi and Ko Sato

Supplementary Figure S1

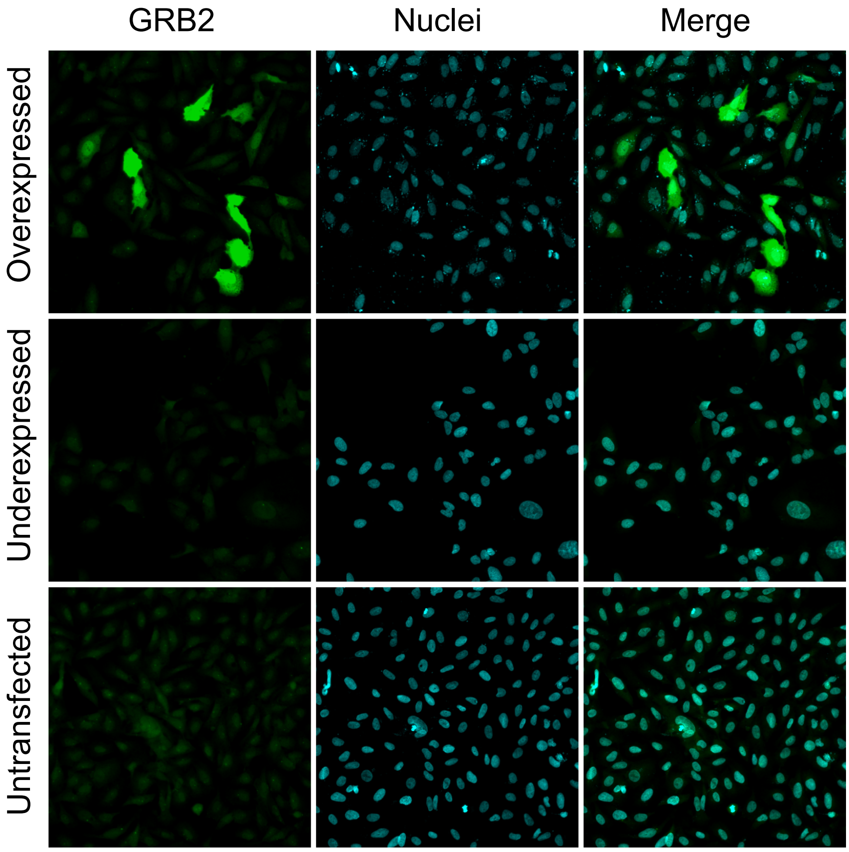

Supplementary Figure S1: Validation of anti-GRB2 antibody. U2OS cells were transfected with GRB2 expression plasmid or antisense oligo directed against GRB2. Forty-eight hours after transfection, cells were stained with anti-GRB2 antibody(green). Nuclei were stained with Hoechest 33342 (1:1000, Invitrogen) (blue). Representative images are shown.

Supplementary Figure S2

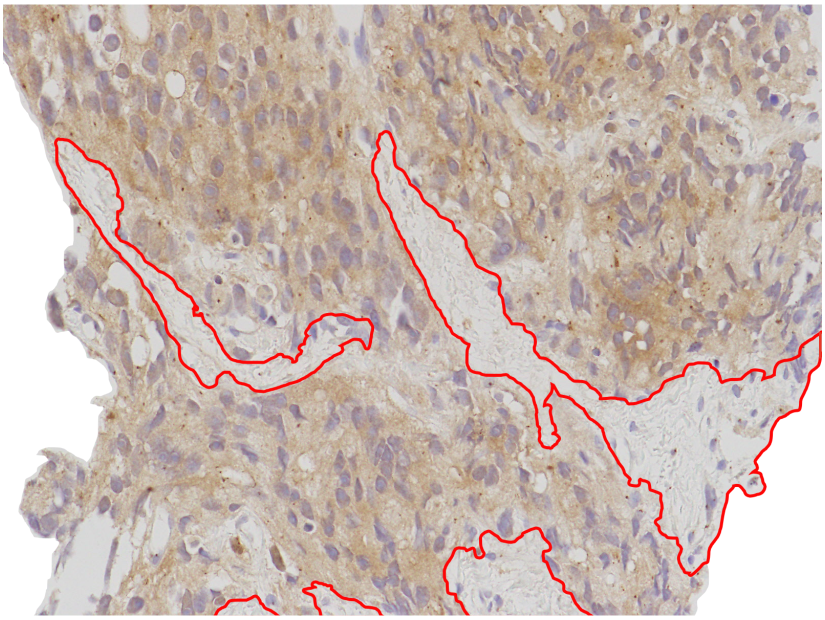

		Cancer	Stroma
Negative		45.396%	94.475%
Positive		54.604%	5.525%
	Strong	0.001%	0%
	Moderate	0.244%	0.049%
	Weak	54.359%	5.476%

Supplementary Figure S2: Validations of protein expression measurements. A representative image of division between cancer and stroma (surrounded by a red line) is shown (top panel). GRB2 expression is quantified in either cancer cells or in stromal cells. Quantified GRB2 values are indicated as percentage of total staining in the table.

Supplementary Figure S3

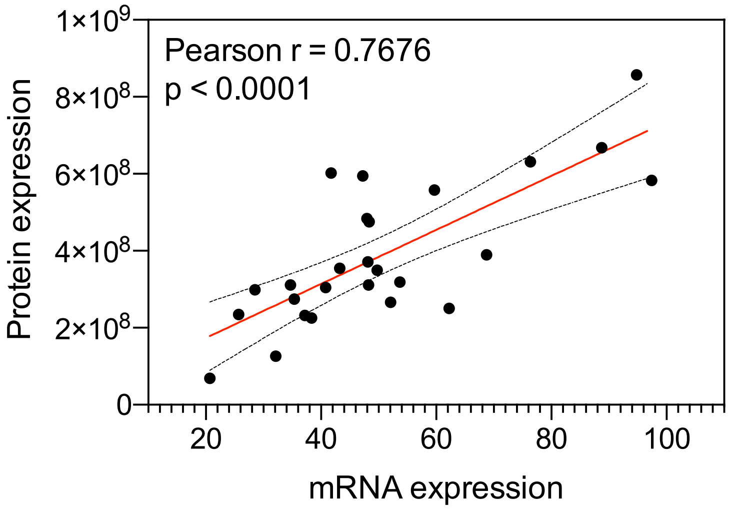

Supplementary Figure S3: Correlation between mRNA and protein expression of GRB2. The x axis indicates mRNA expression as median value of fragments per kilobase of exon model per million mapped reads (FPKM). The Y axis indicates protein expression as median value of normalized iBAQ value.

Supplementary materials and methods

Figure S1. U2OS cells were cultured in DMEM medium supplemented with 10% FBS, 1% Pen/step at 37C in 5% co2 atmosphere. The GRB2 expression plasmid was constructed using VECTORBUILDER. Cells were transfected using X-treme GENE HP (SIGMA) following the manufacture's protocol. The sequences of oligo are GRB2 antisense oligo: 5'-ATATTTGGC GATGGCTTC-3'. To form antisense-lipoplex nanoparticles, a novel cationic liposome system, LipoTrust EX Oligo (Hokkaido System Science, Hokkaido, Japan) was used. The cells were incubated with oligo-lipoplex nanoparticles. Forty-eight hours after transfection, cells were fixed with 4% PFA in PBS for 15 minutes. Then cells were washed with PBS and permeabilized with 0.2% Triton-X100 for 5 minutes and blocked with 3% BSA in PBSt for 15 minutes. Followed by blocking, cells were incubated with the primary antibody for 1 hour at room temperature, then cells were washed three times with PBSt followed by incubated with secondary antibodies for 30 minutes at room temperature.

Figure S3. The dataset (EV1 and EV2) was downloaded from https://www.ncbi.nlm.nih.gov/pmc/articles/PMC6379049/. The expression values (FPKM: mRNA) and normalized iBAQ values (protein) were used. The correlation was analyzed by Pearson correlation in the GraphPad Prism.

Supplementary Table S1

Supplementary Table S1 is available as Supplementary Table S1.txt
